# Innovative exercise device for the abdominal trunk muscles: An early validation study

**DOI:** 10.1371/journal.pone.0172934

**Published:** 2017-02-24

**Authors:** Satoshi Kato, Hideki Murakami, Anri Inaki, Takafumi Mochizuki, Satoru Demura, Junsuke Nakase, Katsuhito Yoshioka, Noriaki Yokogawa, Takashi Igarashi, Naoki Takahashi, Noritaka Yonezawa, Seigo Kinuya, Hiroyuki Tsuchiya

**Affiliations:** 1 Department of Orthopaedic Surgery, Kanazawa University, 13–1 Takara-machi, Kanazawa, Japan; 2 Department of Nuclear Medicine/Biotracer Medicine, Kanazawa University, 13–1 Takara-machi, Kanazawa, Japan; 3 Kanazawa Advanced Medical Center, 13–1 Takara-machi, Kanazawa, Japan; Universite de Nantes, FRANCE

## Abstract

**Background:**

Exercise is one of the few treatments that provide significant improvements in chronic low back pain (CLBP). We developed an innovative exercise device for abdominal trunk muscles. This device can be used in a sitting or standing position and contains a built-in system to measure abdominal trunk muscle strength. We examined whether subjects can adequately use the device to perform the exercises and measure their abdominal trunk muscle strength.

**Methods:**

We collected data on the body height, body weight, body mass index, and girth of 30 healthy male volunteers, and measured their grip power and trunk extensor muscle strength using a dynamometer. The volunteers performed a sit-up test as an indicator of trunk flexor muscle strength, and we measured their abdominal muscle strength using the device. We then evaluated the correlations between abdominal trunk muscle strength and anthropometric parameters as well as the strength of other muscles. In subsequent tests, 5 of the 30 subjects participated in two positron emission tomography (PET) series consisting of examinations after a resting period (control study) and during exercise (exercise study). For the exercise study, the subjects performed 2 sets of exercises for 20 minutes using the device before and after an injection of ^18^F-fluorodeoxyglucose (FDG). PET-computed tomography images were obtained 60 minutes after FDG injection in each study. We compared the skeletal muscle metabolism of the participants in both studies using the standardized uptake value.

**Results:**

The muscle strength measured by the device and the 30-second sit-up frequency were correlated. FDG accumulation within the diaphragm and abdominal rectus muscles was significantly higher in the exercise study.

**Conclusion:**

Our innovative exercise device facilitates a coordinated contraction of the abdominal trunk muscles at the anterior aspect and the roof of the core, and enables subjects to measure the strength of these muscles.

## Introduction

Low back pain (LBP) is a common clinical problem and has significant adverse socioeconomic implications [1]. Approximately 80% of people experience LBP at some point in their life [2, 3]. Nonspecific LBP is defined as LBP not attributable to a recognizable, known specific pathology such as infection, tumor, fracture, structural deformity, inflammatory disorder, or neurological syndrome [4, 5]. This is the most common type of LBP [4, 5]. It has a recurrent course in the majority of patients [6]. Choosing among the huge number of available therapies for LBP could be overwhelming for many specialists, patients, health institutions, and financial institutions [7–10].

Exercise is one of the few treatments that provide significant improvement in chronic LBP (CLBP), and is frequently recommended [5, 9–14]. Exercise takes a longer time to relieve pain than medication or injection. In addition, individual success is notoriously variable and may depend on the patient’s adherence to the prescribed exercise regimen. Adherence to exercise in patients with CLBP may be particularly relevant, especially for the elderly. Elderly individuals with significant CLBP often experience loss of flexibility and/or deformity in the spine, or muscle weakness in the trunk and/or the extremities [15, 16]. Therefore, they may be unable to continue exercising, potentially leading to increased pain. These problems have a negative effect on patient adherence to exercise for CLBP [17], reducing the effect of further exercise. For patients with CLBP, motivation may be especially reinforced if exercise results in substantial reduction of pain and improved function. Other important factors that can affect adherence and motivation include the ability to perform the exercise easily and continuously, and to recognize the effect sooner with achievable short-term goals [17].

**Fig 1:**
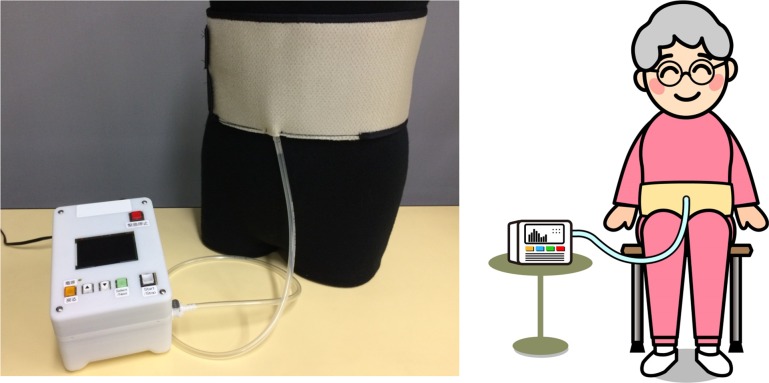
Innovative exercise device for the abdominal trunk muscle. (left) photograph of the device. (right) illustration of a device-equipped subject.

We developed an innovative exercise device for the abdominal trunk muscles (Fig 1: trunk muscle exercise device for research, manufactured by Nippon Sigmax Co., Ltd, Shinjuku-ku, Tokyo, Japan). This device enables patients to perform strengthening exercises for the abdominal trunk muscles in a sitting or standing position without requiring movement of the painful lower back. Therefore, the prescribed exercise is more easily accessible to patients suffering from loss of flexibility, deformity in the spine, or severe pain. The device also contains a built-in system for measuring abdominal trunk muscle strength, which provides an advantage in reinforcing adherence to the exercise unrivaled by existing treatment options. This study aimed to examine whether abdominal trunk muscle strength can be easily measured and whether healthy subjects can adequately perform the muscle strengthening exercises using the device.

## Materials and methods

### Ethics statement

This study was approved by the ethics committee of Kanazawa University Hospital and Kanazawa Advanced Medical Center, and written informed consent was obtained from each subject.

### Description of the device

#### 1. Measurement of abdominal trunk muscle strength

**Fig 2:**
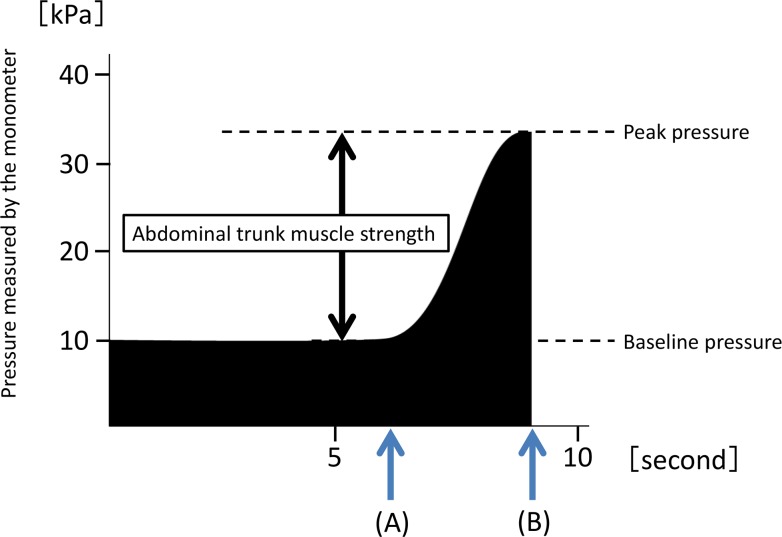
A time course of pressure value indicated by the mechanical manometer of the device during measurement of abdominal trunk muscle strength. (A) indicates the time point when the subject’s abdominal muscles begin to contract against the pressure. (B) shows the reduction in the pressure in the cuff after attainment of the peak pressure.

The device has a similar design to that of a sphygmomanometer, with an inflatable cuff and a mechanical manometer to measure pressure. For the measurement, the cuff is placed around the subject’s abdomen, and then pressure is gradually applied to the abdominal wall. An electrically operated pump is used to inflate the cuff until adequate resistance is encountered from the abdominal muscles. The pressure value indicated on the manometer before measurement is defined as the baseline pressure (Fig 2A). The magnitude of the baseline pressure is set based on the subject’s preference. At the baseline pressure, the subject exerts the maximum force possible over several seconds by contracting the abdominal muscles. The pressure in the cuff is elevated as a result and reaches a peak (the peak pressure). After the pressure reaches a peak, it decreases automatically when the air in the cuff is released. The mechanical manometer calculates and indicates a pressure value that subtracts the baseline pressure from the peak pressure as the value of muscle strength (Fig 2B). The value of muscle strength is defined as that of abdominal trunk muscle strength in this study.

#### 2. Training mode for abdominal trunk muscle strength

**Fig 3:**
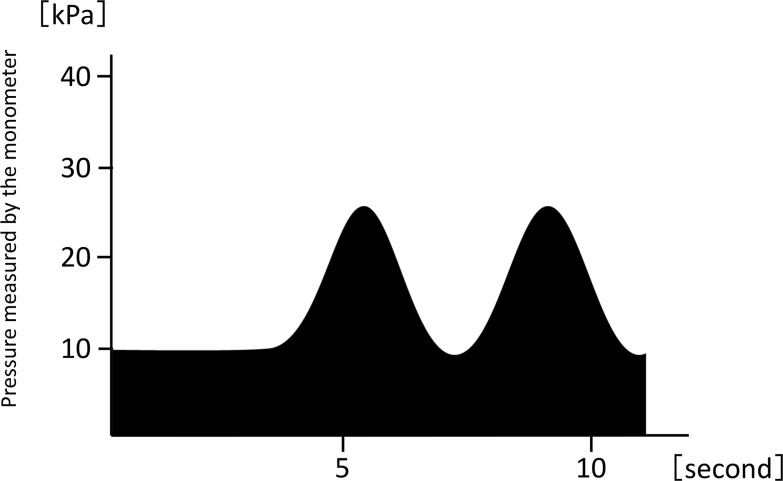
A time course of the pressure value indicated by the mechanical manometer in the device while in training mode for abdominal trunk muscle strength. Under pressure from the cuff, a subject contracts the muscles of the abdominal wall. This force is exerted intermittently at a comfortable pace and strength, for as long a duration as the subject prefers.

After the cuff is placed around the abdomen, an adequate amount of pressure is applied through the cuff. Under the pressure from the cuff, a subject contracts the muscles of the abdominal wall intermittently or continually (Fig 3). This exercise is similar to bracing exercises and functions as a stabilization exercise [18]. However, the exercise is performed under pressure from the cuff. This condition allows subjects to easily and powerfully contract the abdominal muscles.

### Study 1: Evaluation of muscle strength measurement by using the device

Thirty healthy men with a mean age of 31 years (range, 24–42 years) without LBP volunteered for this study. We obtained anthropometric measurements including body height, body weight, body mass index, and girth. We measured the participants’ grip power and trunk extensor muscle (back muscle) strength using a dynamometer and performed a sit-up test to measure the 30-second sit-up frequency as an indicator of trunk flexor muscle strength [19]. In addition, we measured the abdominal trunk muscle strength using our device. We evaluated the correlations between the abdominal trunk muscle strength and anthropometric measurements, as well as the strength of other muscles.

### Study 2: Evaluation of muscle strengthening training by using the device

Five of the 30 men underwent positron emission tomography (PET) scanning with ^18^F-fluorodeoxyglucose (FDG) to examine muscle activity during muscle strengthening exercises using the device. They participated in two PET series consisting of an examination after a resting period (control study) and during exercise (exercise study) to evaluate muscle activity induced by the exercise. Muscle activity during exercise has been examined in previous studies using PET scans [20–23]. FDG taken up by muscle cells is not metabolized and remains in the cells as FDG-6-phosphate after phosphorylation. Thus, FDG accumulation in the muscle can be used as an indicator for glucose intake by the muscle as well as the muscle activity level. The glucose metabolism measured by FDG-PET shows a high correlation with the intensity of muscle activity, and its reliability as an indicator for measuring the amount of muscle activity has been confirmed [24, 25].

None of the subjects was taking any medications, and all were healthy as judged using their medical history and physical examinations. All subjects refrained from eating and drinking for at least 6 hours before the examination as well as strenuous physical activity for at least 1 day before the experiment. For the control study, the participants underwent PET-computed tomography (PET-CT) scanning after rest. After 37 MBq of FDG was injected intravenously in a sitting position, the subjects remained seated until the examination started. For the exercise study, the participants performed muscle strengthening exercises using the device for 20 minutes, followed by an injection of FDG. Immediately after the injection, each subject exercised again for 20 minutes, and then stayed seated until the examination started. In the PET scan examinations, participants were placed in a supine anatomical position on a scanner bed that facilitated longitudinal displacement into the gantry of a PET-CT system (Discovery PET/CT 690; GE Healthcare, Milwaukee, WI, USA). PET-CT images were obtained 60 minutes after the FDG injection in each study. The plasma glucose level of each subject was confirmed to be normal before the FDG injection.

Scanning was performed with a 60-cm axial field of view and a transaxial resolution of 4.9 mm (full-width at half-maximum [FWHM] in the center field of view without scattering medium). Before emission scanning, an unenhanced CT scan was performed for attenuation correction and muscle orientation. Emission scanning was performed in the three-dimensional mode 50 minutes after FDG administration at 3 minutes per bed station. The total emission time was 39 to 42 minutes. Images were reconstructed with three-dimensional ordered subset expectation maximization, with two iterations and 16 subsets. After reconstruction, a 6.4-mm FWHM Gaussian post-filter was applied.

### PET analysis

Regions of interest (ROI) were manually segmented in 14 skeletal muscles located in four transaxial areas of the body: (1) the upper trunk, between the tenth and the twelfth thoracic vertebra for the diaphragm; (2) the lower trunk, around the fourth lumbar vertebra for the abdominal rectus, abdominal external oblique, abdominal internal oblique, transverse abdominal, multifidus, and greater psoas muscles; (3) the pelvis, at the superior border level of the acetabular roof for the gluteus maximus as well as at the gluteus medius, piriformis, obturator internus, and levator ani muscles; (4) the thigh, at the center of the inferior border of the femoral lesser trochanter, the femoral condyle for the quadriceps femoris, and biceps femoris muscles. Plain CT images were used to identify each muscle, and the FDG uptake was evaluated by comparing these CT images with the PET-CT images after a proficient nuclear medicine physician identified and measured the area of all the skeletal muscles.

One experienced nuclear medicine specialist (A.I.) defined the transaxial ROI using plain CT images. The standardized uptake value (SUV) was calculated by overlapping the defined ROI and fusion images. Large vessels were avoided when the muscle areas were outlined. The SUV was calculated to quantitatively examine the FDG uptake of the muscle tissue per unit volume according to the equation: SUV = (mean ROI count [counts per second {cps}/pixel] × calibration factor [cps/Bq])/(injected dose [Bq]/body weight [g]). ROI were defined for the right and left sides of the aforementioned skeletal muscles. The mean SUV was calculated using the following equation: mean SUV = ([left mean SUV × left muscle area] + [right mean SUV × right muscle area])/(left muscle area + right muscle area). We evaluated differences in SUV between the control and exercise studies.

### Statistical analysis

All data are presented as means and standard deviations. For study 1, the Pearson correlation coefficient analysis was used to evaluate the correlations between the muscle strength measured by using the device and the anthropometric parameters as well as the strength of other muscles. Effect size was evaluated with the Cohen r value [26], with r = 0.10 (sample size, 779) being a small effect, r = 0.30 (sample size, 82) being a medium effect, and r = 0.5 (sample size, 26) being a large effect. For study 2, the Wilcoxon signed-rank test was used to evaluate differences in SUV for all the ROIs between the control and exercise studies. All significance levels were set at 0.05. SPSS software version 19.0 for Windows (SPSS Inc., Chicago, Illinois) was used for all statistical analyses.

## Results

### Study 1

**Fig 4:**
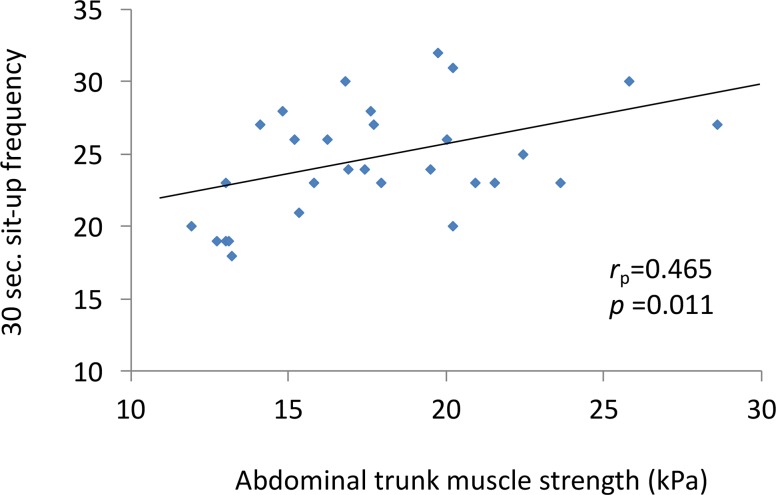
Correlation between the muscle strength values measured using the device and the 30-second sit-up frequency.

Of the 30 men, the mean value of the abdominal trunk muscle strength that was measured by using the device was 17.8 ± 4.1 kPa (range, 11.9–28.6 kPa). Fig 4 shows the correlation between the muscle strength values measured and the 30-second sit-up frequency as an indicator of trunk flexor muscle strength. There was a moderate positive correlation between these two parameters (r_p_ = 0.47, p < 0.05). No significant correlations were observed between the muscle strength values and the anthropometric measurements, or the strength of other muscles (Table 1).

**Table 1:** Correlation between the abdominal trunk muscle strength measured using the device and other parameters (n = 30).

	Mean ± SD	Correlations with abdominal trunk muscle strength	
		R_p_ value	P value
**Abdominal trunk muscle strength measured by the device (kPa)**	17.8 ± 4.1	-	-
**Age (yr)**	30.8 ± 3.9	-0.18	0.34
**Height (cm)**	173.6 ± 4.8	0.19	0.32
**Weight (kg)**	68.1 ± 7.1	-0.06	0.75
**Body-mass index (kg/cm**^**2**^**)**	22.6 ± 2.2	-0.19	0.31
**Abdomen perimeter (cm)**	84.4 ± 6.3	-0.26	0.16
**Grip power (kg)**	46.6 ± 5.3	0.19	0.33
**Back muscle strength (kg)**	111.1 ± 17.1	0.20	0.30
**Sit-up frequency in 30 sec**	24.5 ± 3.8	0.47	<0.05

SD, standard deviation.

### Study 2

**Fig 5:**
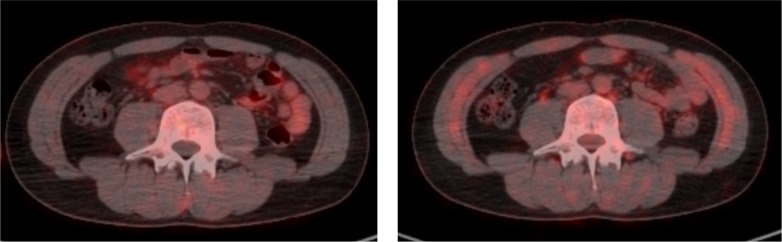
Representative positron emission tomography-computed tomography axial images of the trunk. (Left) the control study (Right) the exercise study.

**Fig 6:**
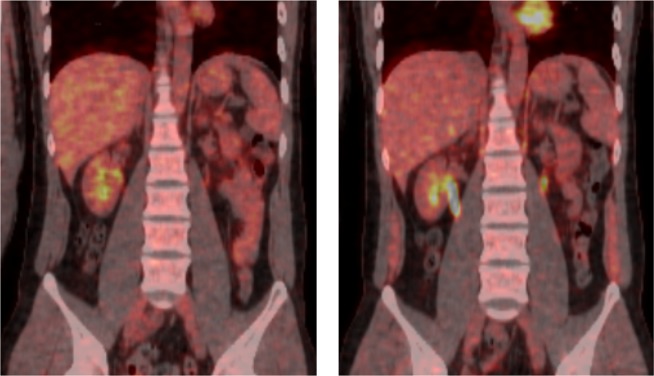
Representative positron emission tomography coronal images of the trunk. (Left) the control study (Right) the exercise study.

Figs 5 and 6 illustrate typical whole-body PET images from the control and exercise studies. Table 2 shows the SUVs of the muscles of participants in both studies. FDG accumulation within the diaphragm and abdominal rectus muscles in the exercise study were significantly higher than those in the control study. No significant differences in mean SUVs were observed between the control and exercise studies for other muscles.

**Table 2:** Mean standardized uptake value in the control and exercise studies (n = 5).

Body area	Muscles	Mean SUVs		P value
		Control study	Exercise study	
Upper trunk	Diaphragm	0.82±0.08	1.22±0.45	<0.05
Lower trunk	Abdominal rectus	0.51±0.13	0.74±0.22	<0.05
	Abdominal external oblique	0.43±0.08	0.48±0.07	0.14
	Abdominal internal oblique	0.56±0.08	0.74±0.29	0.23
	Transverse abdominal	0.58±0.12	0.68±0.20	0.35
	Multifidus	0.80±0.12	0.73±0.04	0.35
	Greater psoas	0.79±0.06	0.80±0.17	0.69
Pelvis	Gluteus maximus	0.56±0.05	0.56±0.09	0.50
	Gluteus medius	0.67±0.05	0.66±0.11	0.50
	Piriformis	0.78±0.13	0.84±0.12	0.35
	Obturator internus	0.85±0.07	0.87±0.21	0.89
	Levator ani	0.77±0.13	0.90±0.18	0.35
Thigh	Quadriceps femoris	0.55±0.03	0.56±0.10	0.50
	Biceps femoris	0.54±0.05	0.53±0.08	0.89

SUV, standardized uptake value.

## Discussion

This study aimed to examine whether we could measure abdominal trunk muscle strength using a novel device and whether participants can adequately perform muscle strengthening exercises using the device. The results of the present study demonstrated that exercise while using the device activated the diaphragm and abdominal rectus muscles. However, no significant differences were observed in most of the parameters tested.

Many forms of exercises have been demonstrated to be effective for improving pain and function in a group of patients with CLBP [9, 10, 27–29]. However, there is no conclusive evidence that one form of exercise is superior to others [11, 27–29]. For every exercise, participant adherence is the most important key to the successful reduction of CLBP. Adherence showed a significant association with the reduction in pain and disability after therapy [30]. Exercises are often tailored to the individual and are performed at home. However, evidence suggests that inadequate adherence to home-based exercises may attenuate the treatment’s efficacy [13, 31, 32]. Adherence to abdominal trunk muscle exercise can be improved using our device for the following reasons: (1) every patient can perform prescribed exercises easily and continuously because exercise using the device does not impose strain on the lower back; and (2) the effect of the exercise can be recognized earlier than with other alternatives, allowing patients to track the improvement of muscle strength. Obtainable short-term goals enable a more pleasant and confidence-inspiring experience, which can motivate patients to continue the training [17], similar to how the measurement of body weight and blood pressure can respectively motivate adherence to dieting and medication.

Recently, there has been a focus on exercises that aim to maintain stability in the lumbar spine [33]. This type of exercise approach has been termed motor control exercise. The core can be described as a muscular box with the abdominals in the front, the paraspinals in the back, the diaphragm as the roof, and the pelvic floor muscles as the bottom [33]. Contraction of the diaphragm increases intra-abdominal pressure, thus adding to spinal stability [34]. Physiotherapists in Queensland highlighted the importance of the deep core musculature, such as the transversus abdominis and multifidi, for core stability [33]. On the other hand, McGill and other investigators emphasized larger “prime mover” muscles, such as the abdominal obliques and quadratus lumborum, in providing spinal stability [35]. It appears that optimal spinal stabilization normally requires a coordinated action or contraction of all deep and superficial core muscles [36]. Muscle contraction under the pressure from the cuff of the device is similar to that in the abdominal bracing exercise proposed by McGill et al [18]. The coordinated contraction of these muscle increases intra-abdominal pressure and spinal stability.

Muscle strength measured using the device was correlated with the strength of the trunk flexors, which include the abdominal rectus and abdominal oblique located in the anterolateral aspect of the abdomen. These muscles are some of the muscles activated by exercise while using the device. Research on core stability exercises has been hampered by a lack of consensus on how to measure core strength. If core instability and core weakness can be measured, outcomes can be determined and proper emphasis can be placed on core strengthening in certain individuals. This device can be a viable option for measuring core muscle strength, and it has the potential to emphasize and improve adherence to exercise.

The limitations of the present study include its small sample size and relatively young volunteers without CLBP. As the aim of the current study was to confirm that subjects could easily use the device, we did not recruit patients with CLBP because the LBP would have significantly reduced their back muscle strength measured using a dynamometer as well as the 30-second sit-up frequency. Therefore, the results of this study cannot be completely adapted to the conditions of patients with CLBP, especially those who are elderly. Further studies with larger cohorts, a wide range of ages, and women are needed to prove the efficacy of the device. Future studies involving patients with CLBP are also needed to validate the efficacy of the device in the treatment of CLBP.

One limitation of the PET study was that the ROI setting and FDG uptake in each skeletal muscle occurred in an arbitrary cross-section; thus, we were unable to study glucose metabolism as a whole for each skeletal muscle. Another limitation was that PET using the FDG method shows only muscle glucose uptake. Other substrates such as lactate, free fatty acids, and muscle glycogen are also metabolized in the active muscle cells but cannot be visualized using this method. Nevertheless, studies have ascertained that glycogen oxidation increases with exercise intensity, and glucose uptake increases with glycogen utilization when exercise intensity increases [37]. In study 2, only two sets of exercises lasting 20 minutes each were performed. The magnitude of the exercises might not be sufficient for the evaluation of muscle activities using PET-CT. Further evaluation will be required to examine whether the other trunk and pelvic muscles, including the abdominal external and internal oblique, transverse abdominal, and pelvic floor muscles, can be activated after continuous exercise for several weeks.

We did not find any limitations to the device in this study. We encountered no difficulties or problems with muscle strength measurement and training. Further studies with the elderly and patients with CLBP are needed to identify drawbacks that may affect the utility of the device.

However, despite the limitations, this study clearly showed that the exercise device facilitates a coordinated contraction of the abdominal trunk muscles at the anterior aspect and the roof of the core, and that the device enables subjects to measure the strength of these muscles.

## Conclusion

The results of the present study indicate that by using our innovative exercise device, subjects can measure the strength of their abdominal trunk muscles and perform exercises that activate the diaphragm and abdominal rectus muscles to stabilize the lumbar spine. Further studies are needed to validate the muscle strengthening effect of exercise using the device as well as the efficacy of the device for the treatment of CLBP.

## Supporting information

S1 Table Data of the abdominal trunk muscle strength, the anthropometric parameters, and the strength of other muscles for all the 30 subjects. (DOCX)

S2 Table Data of the mean standardized uptake values in 14 skeletal muscles in the control and exercise studies for all the 5 subjects. (DOCX)
