# Supplementary material for: Innovative exercise device for the abdominal trunk muscles: An early validation study
Source: PLoS One. 2017 Feb 24;12(2):e0172934. doi: 10.1371/journal.pone.0172934 (PMC5325572; doi:10.1371/journal.pone.0172934)
Supplement: S1 Table — (DOCX) [file pone.0172934.s001.docx]

**Supporting information 1 Table.**

Data of the abdominal trunk muscle strength, the anthropometric parameters, and the strength of other muscles for all the 30 subjects

| Subject No. | Abdominal trunk muscle strength (kPa) | Age (yr) | Height (cm) | Weight (kg) | Body-mass index (kg/cm2) | Abdomen perimeter (cm) | Grip power (kg) | Back muscle strength (kg) | Sit-up frequency in 30 sec. |
| --- | --- | --- | --- | --- | --- | --- | --- | --- | --- |
| 1 | 20.2 | 27 | 171 | 60 | 20.5 | 81 | 46.5 | 114.5 | 20 |
| 2 | 16.8 | 27 | 178 | 71 | 22.4 | 86.5 | 52.7 | 148 | 30 |
| 3 | 21.5 | 27 | 170 | 60 | 20.7 | 74.5 | 37.1 | 104.5 | 23 |
| 4 | 23.6 | 34 | 181 | 68 | 20.7 | 81.5 | 47.5 | 111 | 23 |
| 5 | 17.4 | 32 | 178 | 65 | 20.5 | 80.5 | 51.2 | 144 | 24 |
| 6 | 15.3 | 32 | 175 | 74 | 24.1 | 89 | 48.8 | 103 | 21 |
| 7 | 20 | 29 | 175 | 71 | 23.1 | 87.5 | 50.6 | 130.5 | 26 |
| 8 | 14.1 | 34 | 168 | 65 | 23 | 86.5 | 44.3 | 126 | 27 |
| 9 | 17.9 | 33 | 164 | 59 | 21.9 | 82 | 41.9 | 111.5 | 23 |
| 10 | 13 | 29 | 172 | 63 | 21.2 | 81 | 47.7 | 76.5 | 19 |
| 11 | 15.8 | 30 | 172 | 78 | 26.3 | 99.5 | 41 | 114 | 23 |
| 12 | 28.6 | 32 | 172 | 74 | 25 | 85 | 56.1 | 113 | 27 |
| 13 | 16.9 | 29 | 173 | 77 | 25.7 | 95 | 53.1 | 135 | 24 |
| 14 | 25.8 | 30 | 172 | 64 | 21.6 | 78.5 | 48.4 | 124 | 30 |
| 15 | 20.9 | 37 | 178 | 70 | 22 | 83 | 46 | 124 | 23 |
| 16 | 17.7 | 37 | 172 | 60 | 20.2 | 84 | 43.5 | 112 | 27 |
| 17 | 20.2 | 25 | 179 | 65 | 20.2 | 83 | 45.1 | 118 | 31 |
| 18 | 19.5 | 30 | 177 | 70 | 22.3 | 85.5 | 38.7 | 84.5 | 24 |
| 19 | 20 | 29 | 180 | 80 | 24.6 | 91 | 52.2 | 95.5 | 26 |
| 20 | 13.1 | 31 | 177 | 68 | 21.7 | 87 | 47.8 | 105.5 | 19 |
| 21 | 14.8 | 24 | 173 | 59 | 19.7 | 74 | 43.9 | 104 | 28 |
| 22 | 15.2 | 30 | 170 | 73 | 25.3 | 89 | 48.3 | 130 | 26 |
| 23 | 13 | 33 | 178 | 68 | 21.5 | 81 | 41.2 | 95 | 23 |
| 24 | 12.7 | 29 | 174 | 81 | 26.8 | 94 | 36.3 | 100 | 19 |
| 25 | 16.2 | 29 | 173 | 78 | 26.1 | 95 | 47.4 | 107 | 26 |
| 26 | 19.7 | 31 | 162 | 57 | 21.7 | 80 | 39.4 | 105 | 32 |
| 27 | 13.2 | 42 | 170 | 60 | 20.8 | 79 | 49.9 | 84.5 | 18 |
| 28 | 22.4 | 26 | 180 | 63 | 19.4 | 73 | 44.9 | 91 | 25 |
| 29 | 17.6 | 29 | 178 | 78 | 24.6 | 84 | 57.9 | 120 | 28 |
| 30 | 11.9 | 36 | 166 | 65 | 24.1 | 82 | 47.3 | 101 | 20 |
