# Supplementary material for: Innovative exercise device for the abdominal trunk muscles: An early validation study
Source: PLoS One. 2017 Feb 24;12(2):e0172934. doi: 10.1371/journal.pone.0172934 (PMC5325572; doi:10.1371/journal.pone.0172934)
Supplement: S2 Table — (DOCX) [file pone.0172934.s002.docx]

**Supporting information 2 Table.**

Data of the mean standardized uptake values in 14 skeletal muscles in the control and exercise studies for all the 5 subjects

| Subject No. | 1 | 2 | 3 | 4 | 5 |
| --- | --- | --- | --- | --- | --- |
| Diapharagm Control | 0.813 | 0.794 | 0.933 | 0.71 | 0.857 |
| Diapharagm Exercise | 0.862 | 0.849 | 1.48 | 1.028 | 1.884 |
| Abdominal rectus Control | 0.32 | 0.461 | 0.572 | 0.67 | 0.529 |
| Abdominal rectus Exercise | 0.513 | 0.61 | 1.085 | 0.822 | 0.667 |
| Abd. external oblique Control | 0.334 | 0.388 | 0.54 | 0.477 | 0.4 |
| Abd. external oblique Exercise | 0.379 | 0.471 | 0.562 | 0.452 | 0.543 |
| Abd. internal oblique Control | 0.492 | 0.472 | 0.672 | 0.578 | 0.579 |
| Abd. internal oblique Exercise | 0.593 | 0.768 | 0.49 | 0.603 | 1.228 |
| Transverse abdominal Control | 0.458 | 0.526 | 0.76 | 0.625 | 0.541 |
| Transverse abdominal Exercise | 0.584 | 0.688 | 0.412 | 0.786 | 0.93 |
| Multifidus Control | 0.722 | 0.737 | 0.989 | 0.849 | 0.717 |
| Multifidus Exercise | 0.702 | 0.797 | 0.712 | 0.731 | 0.735 |
| Greater psoas Control | 0.717 | 0.726 | 0.841 | 0.854 | 0.789 |
| Greater psoas Exercise | 0.94 | 0.83 | 0.555 | 0.977 | 0.709 |
| Gluteus maximus Control | 0.484 | 0.578 | 0.611 | 0.56 | 0.559 |
| Gluteus maximus Exercise | 0.586 | 0.6 | 0.393 | 0.62 | 0.597 |
| Gluteus medius Control | 0.591 | 0.681 | 0.681 | 0.739 | 0.648 |
| Gluteus medius Exercise | 0.691 | 0.693 | 0.476 | 0.783 | 0.671 |
| Piriformis Control | 0.608 | 0.747 | 0.939 | 0.879 | 0.741 |
| Piriformis Exercise | 0.931 | 0.79 | 0.697 | 0.984 | 0.8 |
| Obturator internus Control | 0.786 | 0.792 | 0.89 | 0.957 | 0.807 |
| Obturator internus Exercise | 0.976 | 1.124 | 0.608 | 0.932 | 0.727 |
| Levator ani Control | 0.543 | 0.782 | 0.847 | 0.856 | 0.829 |
| Levator ani Exercise | 0.874 | 1.128 | 0.658 | 0.1001 | 0.814 |
| Quadriceps femoris Control | 0.521 | 0.55 | 0.58 | 0.573 | 0.529 |
| Quadriceps femoris Exercise | 0.636 | 0.602 | 0.402 | 0.625 | 0.551 |
| Biceps femoris Control | 0.466 | 0.535 | 0.579 | 0.575 | 0.54 |
| Biceps femoris Exercise | 0.646 | 0.562 | 0.418 | 0.493 | 0.551 |
